# Gonadal Cycle-Dependent Expression of Genes Encoding Peptide-, Growth Factor-, and Orphan G-Protein-Coupled Receptors in Gonadotropin- Releasing Hormone Neurons of Mice

**DOI:** 10.3389/fnmol.2020.594119

**Published:** 2021-01-18

**Authors:** Csaba Vastagh, Veronika Csillag, Norbert Solymosi, Imre Farkas, Zsolt Liposits

**Affiliations:** ^1^Laboratory of Endocrine Neurobiology, Institute of Experimental Medicine, Budapest, Hungary; ^2^Faculty of Information Technology and Bionics, Roska Tamás Doctoral School of Sciences and Technology, Pázmány Péter Catholic University, Budapest, Hungary; ^3^Centre for Bioinformatics, University of Veterinary Medicine, Budapest, Hungary; ^4^Department of Neuroscience, Faculty of Information Technology and Bionics, Pázmány Péter Catholic University, Budapest, Hungary

**Keywords:** GnRH, mouse, proestrus, transcriptome, neuropeptides, growth factors, G-protein- coupled receptors, slice electrophysiology

## Abstract

Rising serum estradiol triggers the surge release of gonadotropin-releasing hormone (GnRH) at late proestrus leading to ovulation. We hypothesized that proestrus evokes alterations in peptidergic signaling onto GnRH neurons inducing a differential expression of neuropeptide-, growth factor-, and orphan G-protein-coupled receptor (GPCR) genes. Thus, we analyzed the transcriptome of GnRH neurons collected from intact, proestrous and metestrous GnRH-green fluorescent protein (GnRH-GFP) transgenic mice using Affymetrix microarray technique. Proestrus resulted in a differential expression of genes coding for peptide/neuropeptide receptors including *Adipor1, Prokr1, Ednrb, Rtn4r, Nmbr, Acvr2b, Sctr, Npr3, Nmur1, Mc3r, Cckbr*, and *Amhr2*. In this gene cluster, *Adipor1* mRNA expression was upregulated and the others were downregulated. Expression of growth factor receptors and their related proteins was also altered showing upregulation of *Fgfr1, Igf1r, Grb2, Grb10*, and *Ngfrap1* and downregulation of *Egfr* and *Tgfbr2* genes. *Gpr107*, an orphan GPCR, was upregulated during proestrus, while others were significantly downregulated (*Gpr1, Gpr87, Gpr18, Gpr62, Gpr125, Gpr183, Gpr4*, and *Gpr88*). Further affected receptors included vomeronasal receptors (*Vmn1r172, Vmn2r-ps54*, and *Vmn1r148*) and platelet-activating factor receptor (*Ptafr*), all with marked downregulation. Patch-clamp recordings from mouse GnRH-GFP neurons carried out at metestrus confirmed that the differentially expressed IGF-1, secretin, and GPR107 receptors were operational, as their activation by specific ligands evoked an increase in the frequency of miniature postsynaptic currents (mPSCs). These findings show the contribution of certain novel peptides, growth factors, and ligands of orphan GPCRs to regulation of GnRH neurons and their preparation for the surge release.

## Introduction

Gonadotropin-releasing hormone (GnRH) plays a key role in the regulation of reproduction (Merchenthaler et al., 1980; Knobil, 1988). This decapeptide is synthesized in neurons of olfactory placode origin that invade the forebrain during ontogenesis and migrate to the sites of their final residence, the medial septum–diagonal band of Broca–medial preoptic area (mPOA). The beaded GnRH axons project—among others—to the median eminence where they discharge their GnRH content into the portal circulation (Merchenthaler et al., 1980) for regulation of the pituitary–gonadal axis (Carmel et al., 1976). The physiological activity, hormone production, and neurohormone release of GnRH neurons are regulated by diverse neuronal circuits of the brain (Spergel, 2019a,b) and by various endocrine hormones and metabolic signals arriving from the periphery (Finn et al., 1998; Smith and Jennes, 2001; Campbell, 2007; Christian and Moenter, 2010; Farkas et al., 2013, 2016; Csillag et al., 2019). The operation of the hypothalamo-pituitary-gonadal (HPG) axis is cyclic including the physiological performance of GnRH neurons (Plant, 2015). Gonadal hormones heavily modulate GnRH neurons and their neuronal afferent systems (Radovick et al., 2012). In female rodents, estradiol (E2) exerts biphasic effects on GnRH neurons and the release of the decapeptide (Sarkar and Fink, 1980; Herbison, 1998). During the ovarian cycle, estradiol principally suppresses the GnRH system via negative feedback mechanisms. The proestrous phase is a functionally important exception when the rising level of E2 restructures the GnRH system together with its coupled regulatory neuronal circuits and prepares them for execution of the forthcoming GnRH surge release (Sarkar et al., 1976; Christian and Moenter, 2010). This positive regulatory feedback mechanism is propelled by E2 acting on estrogen receptors (ERα, ERβ, and GPR30) (Chu et al., 2009; Noel et al., 2009; Terasawa et al., 2009; Moenter and Chu, 2012) expressed in neuronal systems known to regulate reproduction centrally. GnRH neurons are regulated by ERβ (Hrabovszky et al., 2000, 2001), while their neuronal afferent systems are regulated by ERα (Christian et al., 2008; Yeo and Herbison, 2014; Dubois et al., 2015) or both ER subtypes. The positive E2 feedback regulation is known to target GnRH neurons themselves and their distinct regulatory neuron circuits via direct receptor actions (Gore, 2010). Neuronal networks that mediate the negative and positive feedback effects of E2 to GnRH neurons have comprehensively been studied by morphological and functional tools (Wintermantel et al., 2006; Christian and Moenter, 2008; Christian et al., 2008; Yeo and Herbison, 2014; Farkas et al., 2018). GnRH neurons undergo activation in the preovulatory GnRH surge period, characterized by expression of the immediate early gene, c-Fos (Lee et al., 1990), enlarged transcriptional activity (Wang et al., 1995), induction of hormone synthesis (Gore and Roberts, 1997; Finn et al., 1998), and altered firing pattern (Christian et al., 2005; Farkas et al., 2013).

Classic neurotransmitter systems have been found as powerful regulators of GnRH neurons (Smith and Jennes, 2001). The most potential neurotransmitter regulators include gamma-aminobutyric acid (GABA) (Herbison and Moenter, 2011), glutamate (Iremonger et al., 2010), dopamine (DA) (Liu and Herbison, 2013), norepinephrine (NE) (Hosny and Jennes, 1998), serotonin (Bhattarai et al., 2014), acetylcholine (Ach) (Turi et al., 2008), and histamine (H) (Fekete et al., 1999). The expression of genes encoding for neurotransmitter receptors (Todman et al., 2005) and ion channels (Bosch et al., 2013; Norberg et al., 2013; Vastagh et al., 2019) in GnRH neurons has also been verified. In a recent study, we have reported that several neurotransmitter receptors belonging to the aforementioned systems show differential expression in GnRH neurons of proestrous mice (Vastagh et al., 2016). In addition to the classic neurotransmitter systems, neuropeptides, growth factors, and their receptors are equally important regulators of GnRH neurons (Gore, 2010). Electrophysiological studies have provided evidence for direct targeting of GnRH neurons by various peptides via G-protein-coupled receptor (GPCR) signaling mechanisms including anti-Mullerian hormone (AMH) (Cimino et al., 2016; Barbotin et al., 2019), secretin (Csillag et al., 2019), adiponectin (Klenke et al., 2014), alpha-MSH (Roa and Herbison, 2012), AgRP (Roa and Herbison, 2012), CART (Roa and Herbison, 2012), cholecystokinin (CCK) (Giacobini and Wray, 2007), CRH (Phumsatitpong and Moenter, 2018), galanin (Todman et al., 2005), ghrelin (Farkas et al., 2013), GnRH (Todman et al., 2005), glucagon-like peptide 1 (GLP-1) (Farkas et al., 2016), kisspeptin (Pielecka-Fortuna et al., 2008; Pielecka-Fortuna and Moenter, 2010), neuromedin B (Todman et al., 2005), NPY (Roa and Herbison, 2012), somatostatin (Todman et al., 2005), and orexin (Gaskins and Moenter, 2012), among others. In the present study, the proestrus-evoked changes in expression of peptide/neuropeptide-, growth factor-, and orphan GPCR genes of GnRH neurons have been challenged. To achieve this goal, we carried out microarray-based transcriptome analysis of GnRH neurons harvested from regularly cycling, GnRH-green fluorescent protein (GnRH-GFP) transgenic mice at proestrous and metestrous phases of the gonadal cycle. Three of the identified targets were further studied by patch-clamp electrophysiology. The comparative study revealed a differential expression of certain peptide-, growth factor-, and orphan GPCR genes in GnRH neurons of proestrous mice and explored novel regulatory signals and receptors taking part in the regulation of GnRH neurons in proestrus under the positive feedback action of estradiol.

## Materials and Methods

### Animals

Adult, gonadally intact female mice were used from local colonies bred at the Medical Gene Technology Unit of the Institute of Experimental Medicine (IEM). The animals were housed in light-controlled (12:12 light–dark cycle, lights on at 06:00 h) and temperature-controlled (22 ± 2°C) environment, with free access to standard food and tap water. GnRH-GFP transgenic mice (Suter et al., 2000) bred on a C57BL/6J genetic background were used. In this animal model, a GnRH promoter segment drives selective GFP expression in most GnRH neurons. The estrous cycle was monitored daily between 09:00 and 10:00 h by microscopic evaluation of vaginal cytology (Byers et al., 2012). Proestrous (*n* = 6) and metestrous (*n* = 6) female mice with at least two consecutive, regular estrous cycles were used. To avoid the possible circadian effect, animals were sacrificed at the same period of the day, between 16:00 and 18:00 h. Those animals were considered to be in the proestrous stage that fulfilled the following criteria: (1) vaginal smear staining with predominance of nucleated epithelial cells (Byers et al., 2012); (2) luteinizing hormone (LH) serum concentrations >5 ng/ml (15.11 ± 3.4 ng/ml); (3) uterus wet weights >0.15 g (0.19 ± 0.01 g). Accordingly, the following criteria were applied for the metestrous cycle phase: (1) vaginal smears consisting of the three cell types: leukocytes and cornified nucleated epithelial cells (Byers et al., 2012); (2) serum LH levels <0.5 ng/ml (0.35 ± 0.02 ng/ml); (3) uterus wet weights <0.1 g (0.08 ± 0.01 g).

For slice electrophysiological experiments, metestrous mice with uterine weight of <80 mg were used (Silveira et al., 2017).

### Serum Luteinizing Hormone Measurements

Blood samples were collected from the heart of deeply anesthetized mice immediately before the brain fixation step. The samples were chilled on ice, centrifuged at 1,300 g for 3 min at 4°C. Plasma was aspirated, frozen, and stored at −80°C until further use. Serum LH concentrations were measured with a rodent LH ELISA kit #ERK R7010 (assay range: 1–50 ng/ml; sensitivity: 0.5 ng/ml) from Endocrine Technologies Inc. (Newark, CA, USA) according to manufacturers' instructions.

### Laser Capture Microdissection, RNA Isolation, and Whole Transcriptome Amplification

Brain fixation, preparation of sections for the later laser capture microdissection (LCM) and microarray profiling were performed as reported elsewhere (Khodosevich et al., 2007; Vastagh et al., 2015). Briefly, metestrous (*n* = 6) and proestrous female (*n* = 6) mice were deeply anesthetized and perfused transcardially with 80 ml of 0.5% paraformaldehyde followed by 20% sucrose. For microdissection, 7-μm-thick coronal brain sections were cut. Sections were mounted on PEN-membrane slides (Zeiss, Jena, Germany) and processed further for laser microdissection. Uniform and representative sampling of GnRH neurons residing in the mPOA was performed using a PALM MicroBeam system (Carl Zeiss Microimaging GmbH, Jena, Germany), which was equipped with an epifluorescent setup. Sections were cut between coronal planes bregma 0.85 and 0.13 (Paxinos and Franklin, 2012); 250 GFP-positive neurons were dissected and pooled from 80 to 100 consecutive sections of each brain. GnRH neurons were cut precisely along their outlines (plasma membrane) as visualized by the endogenous GFP signal. The collected tissue sample included the perikarya and the short initial segments of the GnRH dendrites.

GnRH cell samples collected with LCM were incubated in 200 ml of lysis buffer at 56°C for 3 h. RNA was isolated from the lysate by proteinase K/acid phenol method. RNA was purified using RNeasy MinElute Cleanup kit (Qiagen, Hilden, Germany). Total RNA was eluted with 14 μl of ribonuclease-free water. The quality of RNA was measured with Bioanalyzer.

Library preparation and amplification were performed according to the manufacturer's (Sigma-Aldrich) instructions for the WTA2 kit. When the SYBR Green signal reached a plateau, the reaction was stopped. The amplified double-stranded cDNA was purified and quantified on a Nanodrop ND-1000 spectrophotometer (Thermo-Fisher Scientific, Waltham, MA, USA).

### Mouse Genome 430 PM Arrays

Eight micrograms of cDNA was fragmented by DNase I and biotinylated by terminal transferase obtained from the GeneChip Mapping 250 K Nsp Assay Kit (Affymetrix Inc., Santa Clara, CA, USA). Hybridization, washing, staining, and scanning of Affymetrix Mouse Genome 430 PM Strip arrays were performed following the manufacturer's recommendations. The Mouse Genome 430 PM Strip array allows the analysis of 34,325 well-annotated genes using 45,123 distinct probe sets. Scanned images (DAT files) were transformed into intensities (CEL files) using the AGCC software (Affymetrix). RMA analysis was performed by the statistical analysis software Partek Genomics Suite (Partek Inc., St. Louis, MO, USA) to obtain probe set level expression estimates.

### Bioinformatics and Data Analysis

All statistical and data mining works were performed in R-environment (R Core Team, 2020) with Bioconductor packages (Huber et al., 2015). Quality assessment of microarrays (*n* = 12) was performed using affyQCReport. Raw microarray data were pre-processed for analysis using RMA (Robust Multi-Array Average) (Irizarry et al., 2003). Fold change (FC) estimation and difference analysis of gene expression were based on linear models combined with Bayesian methods. FC was calculated from normalized and log_2_ transformed gene expression microarray data for each probe sets. The obtained *p*-values were adjusted by the false discovery rate (FDR)-based method. The following cutoff criteria were applied on the differentially expressed gene (DEGs)s: FC > ± 1.5 and adjusted *p* (*p*_adj_) < 0.05.

The differentially regulated genes were displayed in heat map. Kyoto Encyclopedia of Genes and Genomes (KEGG) pathway analysis (http://www.genome.jp/kegg/) was used to reveal the main gene ontology (GO) pathways associated with molecular functions linked to the DEGs. The putative interactions among proteins encoded by DEGs were analyzed by the web-based STRING v11.0 program (https://string-db.org) (Szklarczyk et al., 2015).

### Slice Electrophysiology

Brain slice preparation was carried out as described earlier (Farkas et al., 2010). Briefly, after decapitation, the heads were immersed in ice-cold, low-Na cutting solution, and continuously bubbled with carbogen, a mixture of 95% O_2_ and 5% CO_2_; and the brains were removed rapidly from the skull. The cutting solution contained the following (in mM): saccharose 205, KCl 2.5, NaHCO_3_ 26, MgCl_2_ 5, NaH_2_PO_4_ 1.25, CaCl_2_ 1, and glucose 10. Hypothalamic blocks were dissected, and 250-μm-thick coronal slices were prepared from the mPOA with a VT-1000S vibratome (Leica Microsystems, Wetzlar, Germany) in the ice-cold, low-Na, oxygenated cutting solution. The slices containing preoptic area (POA) were transferred into artificial cerebrospinal fluid (aCSF) (in mM): NaCl 130, KCl 3.5, NaHCO_3_ 26, MgSO_4_ 1.2, NaH_2_PO_4_ 1.25, CaCl_2_ 2.5, and glucose 10, bubbled with carbogen and left for 1 h to equilibrate. Equilibration started at 33°C, and it was allowed to cool down to room temperature.

Recordings were carried out in carbogenated aCSF at 33°C. Axopatch-200B patch-clamp amplifier, Digidata-1322A data acquisition system, and pCLAMP 10.4 software (Molecular Devices Co., Silicon Valley, CA, USA) were used for recording. Neurons were visualized with a BX51WI IR-DIC microscope (Olympus Co., Tokyo, Japan). The patch electrodes (OD = 1.5 mm, thin wall; WPI, Worcester, MA, USA) were pulled with a Flaming-Brown P-97 puller (Sutter Instrument Co., Novato, CA, USA).

GnRH-GFP neurons in the close proximity of the vascular organ of lamina terminalis (OVLT; bregma 0.49–0.85 mm) were identified by brief illumination at 470 nm using an epifluorescent filter set, based on their green fluorescence, typical fusiform shape, and characteristic topography (Suter et al., 2000).

### Reagents and Chemicals

#### Extracellularly Used Drugs

Secretin (30 nM; rat, Tocris) (Csillag et al., 2019); secretin antagonist (3 μM; secretin 5-27; TFTSELSRLQDSARLQRLLQGLV) (Williams et al., 2012); IGF-1 (13 nM; Sigma) (Kleppisch et al., 1992); IGF-1R antagonist JB-1 (800 nM; Bachem, Swiss); neuronostatin-13 (10 nM, rat, mouse, Phoenix Peptide No. 060-48) (Samson et al., 2008).

#### Intracellularly Used Drugs

Membrane impermeable G-protein inhibitor guanosine 5′-[β-thio]diphosphate (2 mM; Meis et al., 2002; Ponzio and Hatton, 2005; Mcdermott and Schrader, 2011; GDP-β-S; Sigma); phosphatidylinositol 3-kinase (PI3K) blocker LY294002 (50 μM, Sigma; Zhang et al., 2016).

### Whole-Cell Patch-Clamp Experiments

Whole-cell patch-clamp measurements started with a control recording (5 min), then the selected receptor ligand was pipetted into the aCSF-filled measurement chamber containing the brain slice in a single bolus, and the recording continued for a further 10 min. Pretreatment with extracellularly applied antagonist started 15 min before adding the ligand and the antagonist was continuously present in the aCSF during the electrophysiological recording. Intracellularly applied membrane impermeable G-protein inhibitor GDP-β-S (2 mM, Sigma; St. Louis, MO, USA) was added to the intracellular pipette solution; and after achieving whole-cell patch-clamp configuration, we waited 15 min to reach equilibrium in the intracellular milieu before starting recording. Each neuron served as its own control when drug effects were evaluated.

The miniature postsynaptic currents (mPSCs) in GnRH neurons were measured as described earlier (Farkas et al., 2010). Briefly, the neurons were voltage clamped at −70 mV of holding potential. Intracellular pipette solution contained the following (in mM): HEPES 10, KCl 140, EGTA 5, CaCl_2_ 0.1, Mg-ATP 4, and Na-GTP 0.4 (pH = 7.3 with NaOH). The resistance of the patch electrodes was 2–3 MΩ. Only cells with low holding current (10 pA) and stable baseline were used. Input resistance (R_in_), series resistance (R_s_), and membrane capacitance (C_m_) were also measured before and after each treatment by using 5 mV hyperpolarizing pulses. To ensure consistent recording qualities, only cells with Rs <20 MΩ, Rin >500 MΩ, and Cm >10 pF were accepted.

Spike-mediated transmitter release was blocked in all mPSC experiments by adding the voltage-sensitive Na-channel inhibitor tetrodotoxin (TTX; 660 nM, Tocris) to the aCSF 10 min before mPSCs were recorded. Time distribution graphs of frequencies were generated using 30 s time bins, shifted by 5 s steps, to show time courses of effect of substances.

To show the effect of agonists and antagonist on the input resistance (R_in_) and capacitance (C_m_) in GnRH neurons, current clamp measurements were recorded. During the measurements, 900-ms-long negative current step was applied (−75 pA). The R_in_ was determined from the voltage response to the application of hyperpolarizing current. The time constant was the time required to reach 63% of the maximum voltage response to hyperpolarizing current (Spergel et al., 1999). The C_m_ was then calculated by dividing the time constant by the R_in_. After control recording, drugs were pipetted into the measurement chamber; and 5 min later, the current step was repeated. In case of intracellularly used blockers, the negative current step was applied immediately after the rupture of the membrane, and it was repeated after 5 min.

### Statistical Analysis

Recordings were stored and analyzed off-line. Event detection was performed using the Clampfit module of the PClamp 10.4 software (Molecular Devices Co., Silicon Valley, CA, USA). The root mean square of the noise was calculated, and then threshold was set at two times the standard deviation of this value, corresponding to the 95% confidence interval. If the amplitude of an mPSC was higher than this threshold level, it was considered as an event.

Spontaneous postsynaptic current (sPSC) and mPSC frequencies were calculated as number of PSCs divided by the length of the corresponding time period (5 or 10 min). Mean values of the control and treated part of the recording are calculated from these frequency values. All the experiments were self-controlled in each neuron: percentage changes in the parameters of the PSCs were calculated by dividing the value of the parameter in the treated period with that of the control period.

Group data were expressed as mean ± standard error of mean (SEM). Two-tailed Student's *t*-test was applied for comparison of groups, and the differences were considered as significant at *p* < 0.05.

## Results

In this study, we examined the impact of proestrus on the expression of peptide/neuropeptide-, growth factor-, and orphan GPCRs in GnRH neurons dissected from intact, metestrous and proestrous GnRH-GFP transgenic mice brains, respectively.

**Figure 1:**
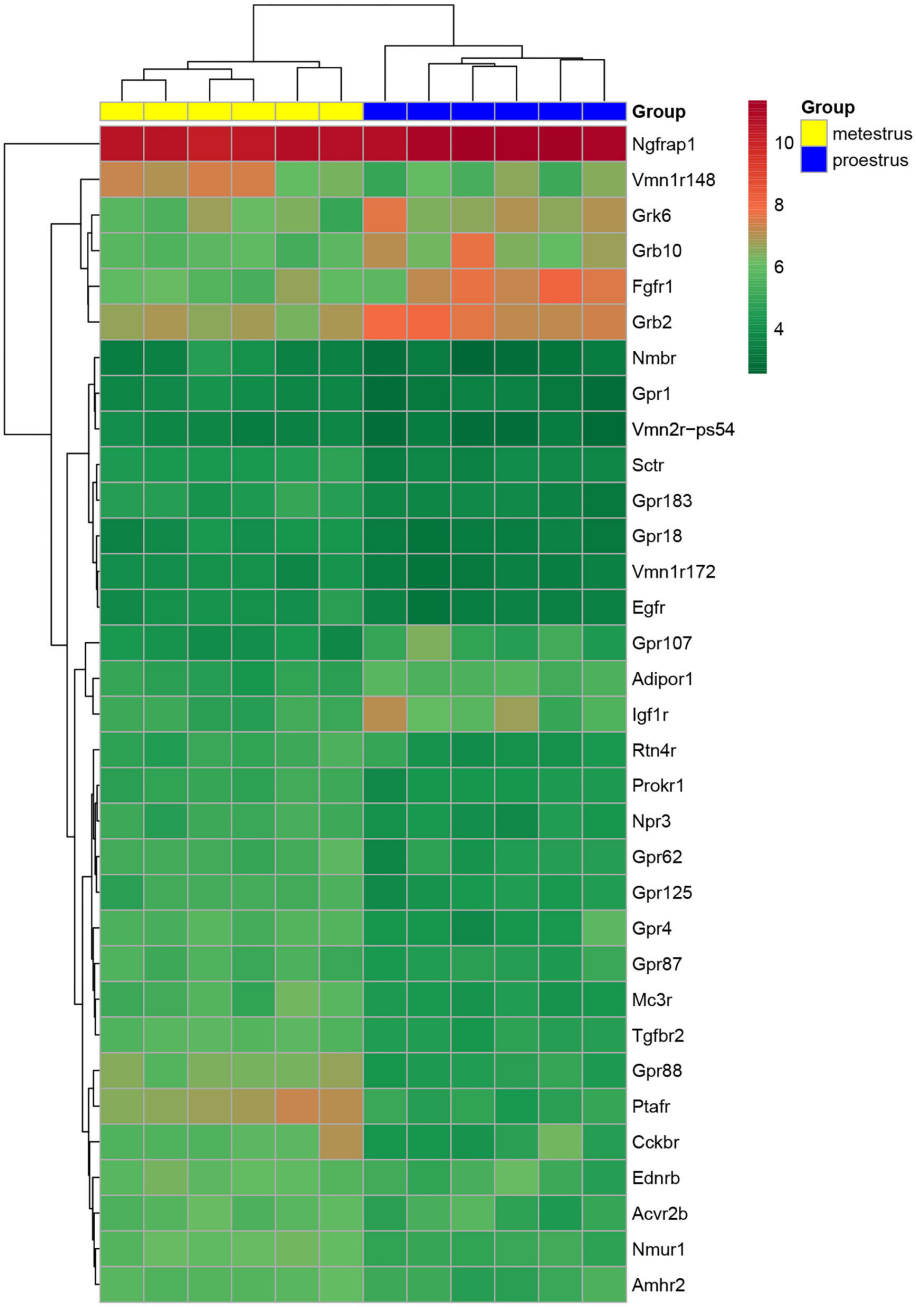
Heat map of peptide/neuropeptide, growth factor, and orphan G-protein-coupled receptor genes regulated differentially in GnRH neurons of proestrous vs. metestrous mice. Expression levels of involved in signaling mechanisms. The rows represent differentially expressed probe sets with corresponding gene symbols on the right. The expression level of each probe is color coded. For decoding, see the color key. The individual samples are shown as columns. The six metestrous and six proestrous samples are coded in yellow and blue, respectively.

**Figure 2:**
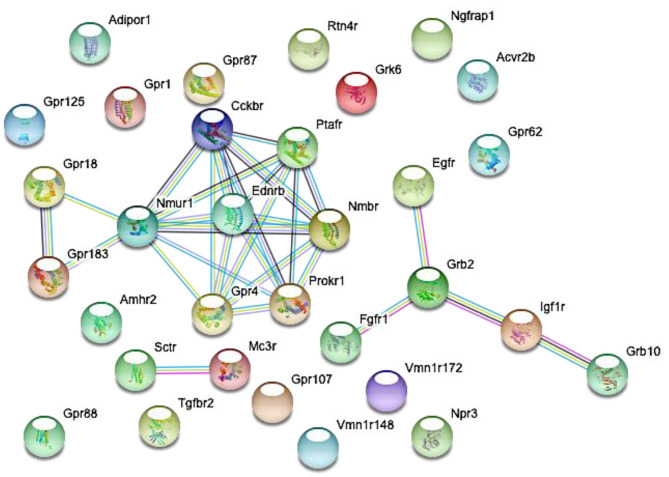
Predicted interactions among proteins encoded by differentially expressed genes in GnRH neurons of intact proestrous mice. The gene network was constructed by using the STRING 10.5 Known and Predicted Protein-Protein Interactions program (http://string-db.org/). Analysis was performed at confidence value of 0.7, and non-interacting elements were also visualized. There are three gene clusters with interacting elements: (1) *growth factor receptors*: Egfr, Fgfr1, Grb2, Igf1r, and Grb10. (2) *Peptide and GPCR receptors:* GPR18, Nmur1, Ptafr, Ednrb, Prokr1, Nmbr, Gpr4, and Cckbr. (3) *Peptide receptors:* Sctr and Mc3r.

Proestrus evoked differential expression of 33 genes in the studied categories. Eight of them were upregulated (Table 1). The differential expression of individual genes was displayed in heat map (Figure 1). The top 10 GO “molecular function” pathways linked to the DEGs are summarized in Table 2. The predicted interactions among proteins encoded by the DEGs in GnRH neurons of late proestrous mice are depicted in Figure 2.

**Table 1:** Differentially expressed genes encoding peptide-, growth factor-, and orphan GPCR receptors in GnRH neurons.

**Probe**	**Symbol**	**Description**	**FC**	**Adj. *p*-val**
**Peptide/neuropeptide signaling**				
1439017_x_at	Adipor1	Adiponectin receptor 1	**2.183**	3.764E−03
1450279_at	Prokr1	Prokineticin receptor 1	0.616	3.905E−03
1423594_a_at	Ednrb	Endothelin receptor type B	0.614	4.199E−02
1419732_at	Rtn4r	Reticulon 4 receptor	0.608	2.452E−02
1422342_at	Nmbr	Neuromedin B receptor	0.605	4.304E−02
1419140_at	Acvr2b	Activin receptor IIB	0.604	3.325E−02
1443454_at	Sctr	Secretin receptor	0.585	1.078E−03
1450286_at	Npr3	Natriuretic peptide receptor 3	0.539	1.787E−03
1421667_at	Nmur1	Neuromedin U receptor 1	0.475	1.851E−04
1422237_at	Mc3r	Melanocortin 3 receptor	0.447	2.462E−03
1460663_at	Cckbr	Cholecystokinin B receptor	0.443	3.521E−02
1457021_x_at	Amhr2	Anti-Mullerian hormone type 2 receptor	0.218	4.651E−05
**Growth factor signaling**				
1425911_a_at	Fgfr1	Fibroblast growth factor receptor 1	**2.456**	1.937E−02
1452108_at	Igf1r	Insulin-like growth factor I receptor	**2.036**	3.486E−02
1449111_a_at	Grb2	Growth factor receptor-bound protein 2	**1.768**	7.138E−03
1430164_a_at	Grb10	Growth factor receptor-bound protein 10	**1.746**	3.595E−02
1428842_a_at	Ngfrap1	Nerve growth factor receptor (TNFRSF16) associated protein 1	**1.525**	7.502E−03
1454313_at	Egfr	Epidermal growth factor receptor	0.478	7.784E−05
1425444_a_at	Tgfbr2	Transforming growth factor, beta receptor II	0.447	4.651E−05
**Signaling via orphan GPCRS**				
1454828_at	Gpr107	G-protein-coupled receptor 107	**1.962**	3.282E−02
1437436_s_at	Grk6	G-protein-coupled receptor kinase 6	**1.933**	4.593E−02
1460123_at	Gpr1	G-protein-coupled receptor 1	0.661	2.011E−02
1420364_at	Gpr87	G-protein-coupled receptor 87	0.618	7.500E−03
1439141_at	Gpr18	G-protein-coupled receptor 18	0.606	6.733E−03
1457236_at	Gpr62	G-protein-coupled receptor 62	0.539	9.132E−03
1444300_at	Gpr125	G-protein-coupled receptor 125	0.524	1.954E−03
1437356_at	Gpr183	G-protein-coupled receptor 183	0.521	1.211E−03
1457745_at	Gpr4	G-protein-coupled receptor 4	0.478	2.166E−02
1460327_at	Gpr88	G-protein-coupled receptor 88	0.296	7.998E−05
**Other signaling mechanisms**				
1421764_at	Vmn1r172	Vomeronasal 1 receptor 172	0.630	3.250E−03
1450274_at	Vmn2r-ps54	Vomeronasal 2, receptor, pseudogene 54	0.628	6.571E−03
1450315_at	Vmn1r148	Vomeronasal 1 receptor 148	0.445	3.759E−02
1427871_at	Ptafr	Platelet-activating factor receptor	0.240	2.202E−05

*The list shows differentially expressed genes (DEGs) involved in peptide, growth factor, and orphan GPCR signaling mechanisms. FC values indicate the changes of expression in the proestrous vs. metestrous GnRH neurons. Gene symbols and FC values of upregulated genes are in bold. FC, fold change; adj. p-val, adjusted p-value*.

**Table 2:** List of the top 10 GO molecular function pathways affected by differentially regulated genes (33) in GnRH neurons of proestrous mice.

**Pathway ID**	**Pathway description**	**Count in gene set**
**Molecular function (GO)**		
GO:0004888	Transmembrane signaling receptor activity	22
GO:0038023	Signaling receptor activity	21
GO:0004930	G-protein-coupled receptor activity	18
GO:0004871	Signal transducer activity	19
GO:0008528	G-protein-coupled peptide receptor activity	7
GO:0001653	Peptide receptor activity	6
GO:0042562	Hormone binding	5
GO:0019199	Transmembrane receptor protein kinase activity	5
GO:0017046	Peptide hormone binding	4
GO:0005026	Transforming growth factor beta receptor activity, type II	2
GO:0019838	Growth factor binding	4
GO:0005057	Receptor signaling protein activity	4
GO:0008188	Neuropeptide receptor activity	3
GO:0043560	Insulin receptor substrate binding	2
GO:0042277	Peptide binding	4
GO:0004702	Receptor signaling protein serine/threonine kinase activity	3
GO:0004714	Transmembrane receptor protein tyrosine kinase activity	3
GO:0016500	Protein-hormone receptor activity	2
GO:0004675	Transmembrane receptor protein serine/threonine kinase activity	2
GO:0045028	G-protein-coupled purinergic nucleotide receptor activity	2
GO:0005158	Insulin receptor binding	2
GO:0005070	SH3/SH2 adaptor activity	2
GO:0042923	Neuropeptide binding	2

*Transmembrane signaling receptor activity, signaling receptor activity, G-protein-coupled receptor activity, and signal transducer activity pathways are represented by the largest count numbers among the gene sets (19–22)*.

### Differential Expression of Genes Encoding Peptide/Neuropeptide Receptors

Analysis of microarray data revealed DEGs associated with various peptidergic signaling mechanisms (Table 1, Figure 1). Twelve G-protein-coupled peptide/neuropeptide receptors showed differential expression in proestrus. Most of them (11 genes) were downregulated. The only upregulated receptor gene was adiponectin receptor 1 (*Adipor1*). The downregulated group of genes involved prokineticin receptor 1 (*Prokr1*), endothelin receptor type B (*Ednrb*), reticulon 4 receptor (*Rtn4r*), neuromedin B receptor (*Nmbr*), activin receptor IIB (*Acvr2b*), secretin receptor (*Sctr*), natriuretic peptide receptor 3 (*Npr3*), neuromedin U receptor 1 (*Nmur1*), melanocortin 3 receptor (*Mc3r*), cholecystokinin B receptor (*Cckbr*), and AMH type 2 receptor (*Amhr2*).

### Effects of Proestrus on Expression Profile of Growth Factor Receptors

Like peptide receptors, the expression of growth factor receptors and their adaptor/associated proteins was altered in proestrus (Table 1, Figure 1). Altogether, seven genes showed differential expression. Fibroblast growth factor receptor 1 (*Fgfr1*) and insulin-like growth factor I receptor (*Igf1r*) genes were upregulated. The expression of growth factor-bound/associated proteins also increased involving growth factor receptor-bound protein 2 (*Grb2*), growth factor receptor-bound protein 10 (*Grb10*), and nerve growth factor receptor (TNFRSF16) associated protein (*Ngfrap1*). Epidermal growth factor receptor (*Egfr*) and transforming growth factor beta receptor II (*Tgfbr2*) genes showed downregulation.

### Changes in Expression of Orphan G Protein Receptor-Coupled Receptors in Proestrus

Proestrus had a profound effect on the expression of orphan GPCRs (Table 1, Figure 1) by upregulating GPR107 and downregulating eight members of the receptor family (*Gpr1, Gpr87, Gpr18, Gpr62, Gpr125, Gpr183, Gpr4*, and *Gpr88*). A GPCR kinase (*Grk6*) also showed upregulation.

### Differential Expression of Other Peptide Receptors

In this group, genes encoding a few vomeronasal receptors (*Vmn1r172, Vmn2r-ps54*, and *Vmn1r148*) and platelet-activating factor receptor (*Ptafr*) were affected. All of them showed decreased expression in proestrus (Table 1, Figure 1).

### Operability of Differentially Expressed Receptors in Gonadotropin-Releasing Hormone Neurons

**Figure 3:**
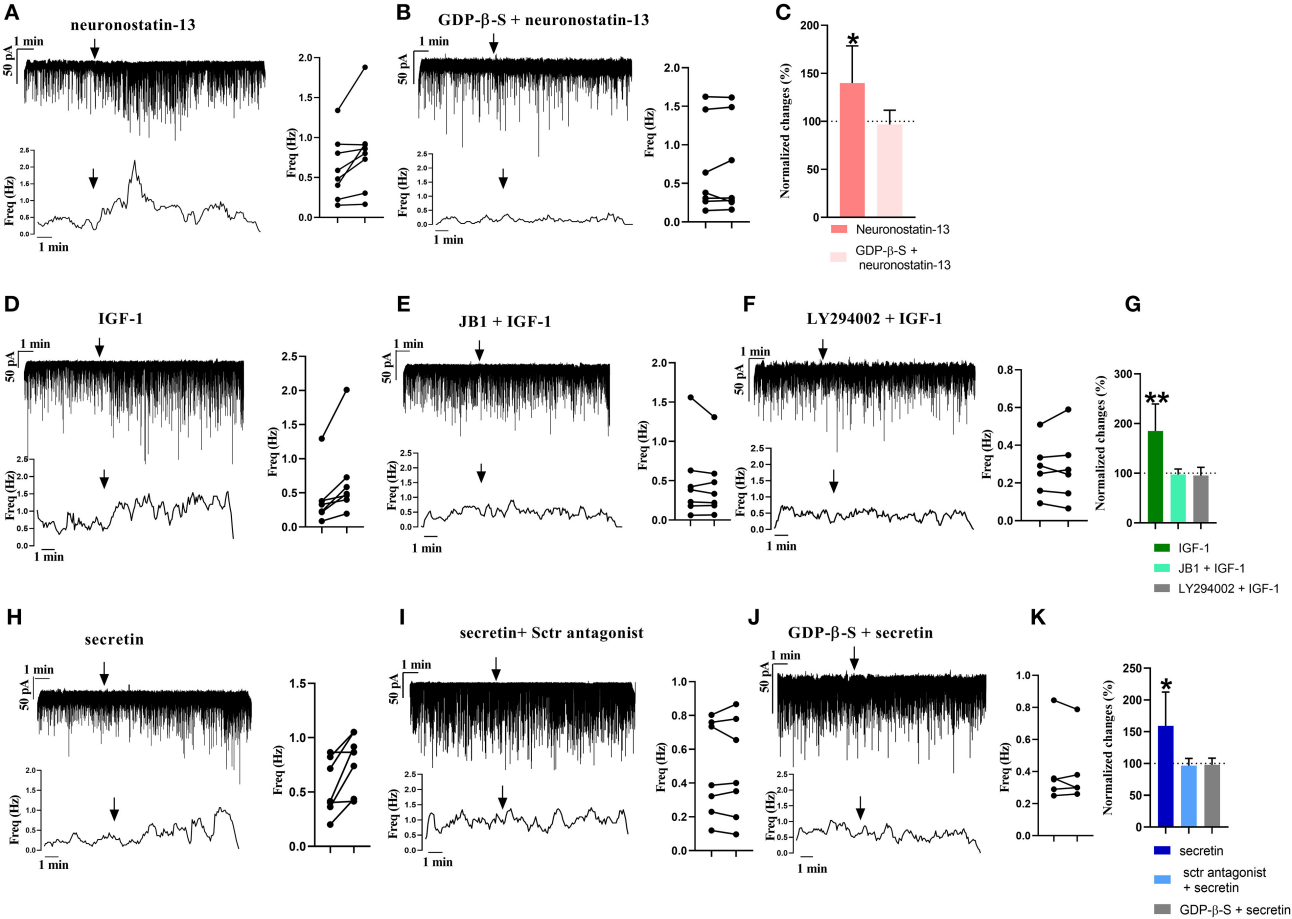
Electrophysiological validation of functional peptide/growth factor receptors expressed in GnRH neurons of metestrous mice. Changes in the frequency of mPSCs after administration of different substances. Under each representative recording, the corresponding frequency distribution graph is shown. On the right, the control and treated mPSC frequency of each cell is displayed individually (paired *t*-test *p < 0.05). Arrow shows the application of the drugs. **(A)** Significant increase in the mPSCs is observed after neuronostatin treatment. **(B)** Application of intracellular GDP-β-S prevented the effect of neuronostatin-13. **(C)** Bar graph shows the normalized changes after neuronostatin-13 and antagonist treatment. Control period was considered as 100% (Student's *t*-test; *p < 0.05). **(D)** IGF-1 increases frequency of mPSCs. **(E)** JB1 abolished the effect of IGF-1. **(F)** In the presence of intracellularly applied PI3K blocker LY294002, IGF-1 application did not elevate the frequency. **(G)** Bar graph shows the normalized changes after IGF-1 application on mPSCs, and after the IGF-1 receptor antagonist JB1 or intracellularly used Pi3K blocker LY294002 treatment. Control period was considered as 100% (Student's *t*-test; **p < 0.01). **(H)** Secretin elevated the frequency of mPSCs. **(I)** Sctr antagonist prevented the effect of secretin. **(J)** Effect of secretin in the presence of intracellularly used GDP-β-S. **(K)** Bar graph shows the normalized changes after the applied treatments. Control period was considered as 100% (Student's *t*-test; *p < 0.05).

From the three main signaling categories, the functionality of three receptors was examined further by patch-clamp electrophysiology. The selected receptors included GPR107, the putative receptor of neuronostatin, insulin-like growth factor 1 (IGF-1) receptor, and secretin receptor. The passive membrane parameters remained unchanged after administration of the agonists and antagonists (Table 3). Neuronostatin-13 (10 nM) significantly increased the frequency of mPSCs by 39.9% in GnRH neurons compared with the control period (0.6145 ± 0.1388 Hz, Student's *t*-test, *p* = 0.0224), and its effect was totally abolished by intracellularly applied G-protein inhibitor administration (GDP-β-S; 2 mM) (Figures 3A–C). Neuronostatin-13 (10 nM) was not able to change the amplitude of mPSCs (Table 4). Exposure of the preoptic slices to IGF-1 (13 nM) also evoked an increase (by 85%) in the frequency but not in the amplitude of mPSCs compared with the control (0.4164 ± 0.1516 Hz, Student's *t*-test, *p* = 0.0061) (Figures 3D,G, Table 4) in these hypophysiotropic neurons. The graphs also demonstrated that this facilitatory event was prevented by administration of the extracellularly used IGF-1 receptor antagonist, JB1 (800 nM), or intracellularly used PI3K blocker LY294002 (50 μM) (Figures 3E–G, Table 4), prior to the ligand exposure. The bath application of the secretin hormone at 30-nM concentration also augmented the frequency of mPSCs by 59.1% (Figures 3H,K, Table 4), but the amplitude remained stable during the measurements (Table 4). The secretin receptor antagonist, secretin 5-27 (3 μM) or the intracellularly applied G-protein blocker GDP-β-S (2 mM) powerfully blocked this event (Figures 3I–K, Table 4).

**Table 3:** Changes in passive membrane properties after administration of various agonists and antagonists.

**Substance**	**Control period**	**% changes, 5 min after administration**	***p*-value**	***n***	***N***
**Membrane capacitance (C**_**m**_**)**					
Neuronostatin-13	24.58 ± 4.467 pF	97 ± 9.806	0.7797	5	2
Igf-1	25.29 ± 3.912 pF	102.8 ± 4.270	0.5654	4	2
Secretin	22.95 ± 3.418 pF	106.7 ± 8.686	0.523	5	2
JB1	26.28 ± 1.514 pF	101.3 ± 3.756	0.7566	4	2
Secretin receptor antagonist	28.41 ± 0.4295 pF	97 ± 4.163	0.546	5	3
PI3K blocker	28.31 ± 3.815 pF	107 ± 4.726	0.2767	4	2
GDP-beta-S	31.01 ± 2.202 pF	110.7 ± 12.20	0.4741	5	2
**Input resistance (R**_**in**_**)**					
Neuronostatin-13	767.8 ± 108.5 MΩ	95 ± 8.377	0.5926	5	2
Igf-1	732.6 ± 93.21 MΩ	88.25 ± 4.679	0.0869	4	2
Secretin	745.9 ± 30.94 MΩ	109.3 ± 4.333	0.1641	5	2
JB1	836 ± 42.81 MΩ	94.67 ± 1.764	0.0942	4	2
Secretin receptor antagonist	838.1 ± 71.87 MΩ	100.3 ± 5.0444	0.9533	5	3
PI3K blocker	795.3 ± 89.66 MΩ	89.67 ± 14.95	0.5608	4	2
GDP-beta-S	727.3 ± 35.33 MΩ	93 ± 2.309	0.0938	5	2

*n, number of neurons measured; N, number of animals used (Student's t-test)*.

**Table 4:** Changes in mPSC frequency and amplitude of GnRH neurons upon application of different substances.

	**Substance**	**Value of control periods (mean ± SEM)**	**Normalized changes compared with the control 100% (mean ± SEM)**	***p*-value of the normalized changes (Student's *t*-test)**	***n***	***N***
Frequency changes	Neuronostatin-13	0.6145 ± 0.1388 Hz	139.9 ± 13.66	0.0224^*^	8	3
	GDP-β-S + neuronostatin-13	0.6893 ± 0.227 Hz	96.67 ± 6.075	0.6068	6	3
	IGF-1	0.4164 ± 0.1516 Hz	185 ± 20.54	0.0061^**^	6	2
	JB1 + IGF-1	0.4953 ± 0.1908 Hz	97.29 ± 4.224	0.5443	7	2
	secretin	0.5409 ± 0.09737 Hz	159.1 ± 20.16	0.0262^*^	7	3
	Sctr antagonist + secretin	0.4794 ± 0.1063 Hz	96.57 ± 4.303	0.4560	7	2
	LY294002 + IGF-1	0.273 ± 0.059 Hz	95.17 ± 6.819	0.5101	6	2
	GDP-β-S + secretin	0.41 ± 0.108 Hz	101.4 ± 6.345	0.8362	5	2
Amplitude changes	Neuronostatin-13	30.86 ± 9.705 pA	102.9 ± 2.091	0.2045	8	3
	GDP-β-S + neuronostatin	53.38 ± 11.31 pA	103 ± 2.025	0.2126	6	3
	IGF-1	66.94 ± 11.96 pA	104.0 ± 2.121	0.1324	6	2
	JB1 + IGF-1	58.79 ± 9.943 pA	104.1 ± 1.738	0.0545	7	2
	secretin	31.36 ± 5.566 pA	108.9 ± 6.847	0.2434	7	3
	Sctr antagonist + secretin	49.66 ± 10.33 pA	101.4 ± 2.136	0.5286	7	2
	LY294002 + IGF-1	52.77 ± 6.419 pA	104.8 ± 1.973	0.058	6	2
	GDP-β-S + secretin	61.16 ± 12.93 pA	99.8 ± 2.035	0.9264	5	2

*n, number of neurons measured; N, number of animals used*.

* p < 0.05;

** *p < 0.01 (Student's t-test)*.

## Discussion

The main findings of the study reveal that proestrus changes the expression of genes encoding peptide-, growth factor-, and orphan GPCRs in GnRH neurons of mice and confirm the significance of different neuropeptides, growth factors, and ligands of orphan receptors, all acting via GPCRs of GnRH neurons in orchestration of the pre-ovulatory GnRH surge. These events together with classical neurotransmitter signaling mechanisms (Vastagh et al., 2016) and voltage-gated ion channels (Vastagh et al., 2019) contribute to shifting the phenotype of the GnRH neuron from metestrous to proestrous type and to initiation of downstream actions that prime the cells for surge release of GnRH, a hormonal prerequisite of activation of the pituitary–gonadal axis and the subsequent ovulation.

### Methodological Considerations

Estrous cycle-dependent comparative investigations are restricted to the whole hypothalamus at the cost of the lack of cell type-specific spatial resolution (Dicarlo et al., 2017). The list of DEGs in proestrus–metestrus pairwise comparison of mouse hypothalami by DiCarlo et al. do not show any overlap with DEGs of neuropeptide/growth factor and orphan GPC receptors of the GnRH neurons presented in our study. This evidence strengthens the view that the observed differential gene expression is GnRH cell type specific, and it is due to the gonadal cycle.

The rationale behind studying the mPSCs is that postsynaptic actions on GnRH neurons modify the frequency of mPSCs by altering the retrograde endocannabinoid and/or NO signaling in the presynaptic terminals (Farkas et al., 2010, 2013, 2018; Balint et al., 2016; Csillag et al., 2019). In this study, the electrophysiological recordings in the presence of intracellular blockers of the IGF-1 and neuronostatin signaling pathways confirm the postsynaptic action of the peptides.

### Proestrus Modifies the Expression of Peptide/Neuropeptide Receptors

#### Adiponectin Receptor 1

Adiponectin secreted from the adipose tissue is a potent regulator of fatty acid oxidation and glucose utilization. In GT1-7 neurons, both adiponectin receptor 1 and 2 are expressed, and the hormone inhibits GnRH secretion via AMP-activated protein kinase (Wen et al., 2008). In nasal explants of mice, GnRH neurons have been reported to express adiponectin receptor 2 (AdipoR2), and about 20% of the cells responded to adiponectin (Klenke et al., 2014), because this substance evoked hyperpolarization of GnRH neurons and decreased calcium oscillations. In this study, the nasal pits were isolated without regard to the sex of the animal. Our present results show a differential expression of adiponectin receptor 1 in GnRH neurons of proestrous mice, with a marked upregulation of the coding gene. Elucidation of the functional role of adiponectin signaling via AdipoR1 in GnRH neurons during the positive estradiol feedback awaits further studies.

#### Prokineticin Receptor 1

Prokineticin signaling has been extensively studied in the regulation of reproduction (Maldonado-Perez et al., 2007). Failure of this signaling mechanism results in abnormal development of the olfactory bulb and the reproductive system (Dode et al., 2006; Matsumoto et al., 2006). The role of prokineticin 2 and prokineticin receptor 2 has been addressed in processes of reproduction, including the human HPG axis (Pitteloud et al., 2007; Sarfati et al., 2010; Balasubramanian et al., 2011, 2014). Here, we report that GnRH neurons express downregulated prokineticin 1 receptor in late proestrous mice. The main putative source of the ligand is the nucleus of the solitary tract of the adult mouse brain (Cheng et al., 2006). Prokineticin 1 mRNA expression was detected in the olfactory region, dentate gyrus, zona incerta, and dorsal motor vagal nucleus (Cheng et al., 2006). Functional studies are required to dissect further the role of prokineticin-1 receptor in actions of GnRH neurons.

#### Endothelin Receptor Type B

Endothelins have been shown to regulate neurosecretion in immortalized GnRH neurons via their specific receptors (Krsmanovic et al., 1991). Endothelin 1 acting on endothelin receptor B controls the migration of human olfactory GnRH-secreting neuroblasts (Romanelli et al., 2005). Endothelin receptor beta like immunoreactivity was observed in the OVLT and median eminence of the rat brain, with clear association with GnRH axons (Yamamoto et al., 1997). Our data confirm the expression of the receptor in mouse GnRH neurons and prove the differential expression of its coding gene in proestrus.

#### Reticulon 4 Receptor

This receptor binds the myelin-associated protein, Nogo, which inhibits axon outgrowth and regulates neuronal plasticity. Proestrus downregulates its expression.

The physiological significance of this signaling mechanism in case of the GnRH system is still obscure.

#### Neuromedin B Receptor

Neuromedin B receptor expression has already been reported in mouse GnRH neurons with a marked depolarizing effect of its specific ligand (Todman et al., 2005). Intracerebroventricular administration of neuromedin B—by acting at the level of the hypothalamus—increases plasma LH (Boughton et al., 2013). We found the downregulation of neuromedin receptor B in the late proestrous phase of the gonadal cycle.

#### Activin Receptor IIB

Activin is expressed in neurons of the hypothalamus, and activin-IR axons are juxtaposed to GnRH neurons (Macconell et al., 1998). Activin-A has been reported to increase the secretion of GnRH from GT1-7 cells (Gonzalez-Manchon et al., 1991). In male rats, intracerebroventricular administration of activin-A increases the secretion of follicle-stimulating hormone (FSH) and evokes a modest LH release, without changing the GnRH mRNA expression (Lee and Rivier, 1997). In explanted male hypothalamus, activin-A stimulated the GnRH release, and its effect was eliminated by inhibin and blunted by testosterone (Calogero et al., 1998). Activin receptor type II null (Acvr2^−/−^) male mice show altered reproductive behavior with marked deficits in capacity of copulation and ejaculation (Ma et al., 2005). Our present data indicate the presence of activin receptor IIB in mouse GnRH neurons and its differential expression in late proestrus.

#### Secretin Receptor

Our current knowledge about secretin signaling in the brain (Zhang and Chow, 2014) is limited. *In situ* hybridization histochemistry explored the distribution of secretin receptor mRNA-expressing cells in the brain, including the OVLT region (Toth et al., 2013). The release of secretin from the hypothalamus has been reported earlier (Chu et al., 2006). Secretin activates hypothalamic magnocellular neurons with involvement of noradrenergic signaling mechanisms in the rat (Velmurugan et al., 2010). The involvement of secretin signaling in the regulation of GnRH neurons of the male mouse has recently been shown (Csillag et al., 2019). Our current finding raises the possibility of a direct targeting of GnRH neurons by secretin in the female, too, and the estrus cycle phase-dependent nature of the regulation with a manifest downregulated state of secretin receptors in late proestrus. The acquired electrophysiological data indicate that secretin receptors expressed in female GnRH neurons are operational and that their activation by the natural ligand increases the frequency of mPSCs in metestrous mice.

#### Natriuretic Peptide Receptor 3

Natriuretic peptide A and B receptors have previously been described in mouse GnRH neurons (Todman et al., 2005). We report here the presence and differential expression of NPR3 gene encoding natriuretic peptide C receptor in GnRH neurons of proestrous mice. In GT1-7 cell line, natriuretic peptides stimulate cyclic GMP production (Olcese et al., 1994). The role of natriuretic peptides in the central control of reproductive hormone secretion has also been substantiated (Samson et al., 1992). The functional aspects of natriuretic peptide signaling via the C type receptor await clarification.

#### Neuromedin U Receptor 1

This receptor is expressed in 50% of the studied mouse GnRH neuron pools (Todman et al., 2005). The expression of neuromedin U receptor is downregulated in proestrus. Neuromedin U is also synthesized in the hypothalamus, and it controls LH secretion (Vigo et al., 2007). Furthermore, the effect of centrally administered neuromedin U is dependent on the phase of gonadal cycle.

#### Melanocortin 3 Receptor

GnRH neurons receive substantial orexigenic and anorexigenic peptide signals from the arcuate nucleus (Roa and Herbison, 2012). Alpha-melanocyte-stimulating hormone (MSH) regulates GnRH neurons via MC3 and MC4 receptors; and the signaling activates the hypophysiotropic neurons (Roa and Herbison, 2012). Alpha-MSH also stimulates the secretion of GnRH from the GT1-1 cell line (Khong et al., 2001).

#### Cholecystokinin B Receptor

CCK exerts its regulatory role via type 1 (CCK-1R) and 2 (CCK-2R) receptors. The significance of CCK signaling in the development and operation of the GnRH system has already been addressed. Regarding the developmental aspects of the regulation, CCK exerts an inhibitory influence via CCK-1R on migration of GnRH neurons (Giacobini et al., 2004). CCK-IR axons contact GnRH neurons in the mouse brain (Giacobini and Wray, 2007). The hormone induces the activity of GnRH neurons via CCK-1R. Accordingly, in a nasal explant model, antagonization of CCK-1R was found to increase the number of calcium peaks/GnRH neuron, mean peak amplitude, and percentage of GnRH cells exhibiting high activity (Giacobini and Wray, 2007). The present study confirms that adult female GnRH neurons also express CCK-2R, whose expression is differentially regulated in proestrus.

#### Anti-Mullerian Hormone Type 2 Receptor

The powerful regulatory role of AMH via AMH-2R in hypothalamic control of reproduction has recently been discovered (Cimino et al., 2016). Consequently, GnRH neurons express AMH-2R, and AMH activates the firing of GnRH neurons and increases the GnRH-dependent release and pulsatility of LH (Cimino et al., 2016; Barbotin et al., 2019). Insufficient AMH signaling to GnRH neurons interferes with their development and results in hypogonadotropic hypogonadism (Malone et al., 2019). Our present data suggest the participation of AMH signaling in GnRH neurons of proestrous mouse exemplified by downregulation of its receptor at late proestrus.

### Proestrus-Evoked Alterations in Growth Factor Receptor Expression

#### Fibroblast Growth Factor Receptor 1

Basic fibroblast growth factor signaling is vital in the development and regulation of GnRH neurons (Chung et al., 2016). It promotes the emergence of GnRH neurons and increases the neurite outgrowth and arborization in nasal explants (Gill et al., 2004). The growth factor is important to the proper morphogenesis of the olfactory bulb and migration and maturation of GnRH neurons (Hu et al., 2013). Disruption of this signaling contributes to Kallmann syndrome. Type 1 fibroblast growth factor receptor expression was confirmed in immortalized GnRH neurons. Ligand activation of the receptor evokes cell proliferation and enhances the steady-state level of mRNA encoding the GnRH precursor processing endoprotease prohormone convertase 2 (PC2) (Voigt et al., 1996). Fibroblast growth factor 8 signaling via FGFR1 is vital in emergence of GnRH neurons (Chung et al., 2008); its diminution causes GnRH deficiency in humans and mice (Falardeau et al., 2008). Our current finding about the expression and upregulation of FGFR1 in GnRH neurons supports the view that this signaling mechanism is operational in adult female mice in proestrus.

#### Insulin-Like Growth Factor I Receptor

IGF-1 signaling mechanisms regulate the HPG axis at various levels. Its role in reproduction has extensively been studied and reviewed (Daftary and Gore, 2005; Wolfe et al., 2014). Central IGF-1 receptors play a crucial role in the maintenance of the estrus cycle. Administration of IGF-1 receptor antagonist (JB-1) into the ventricular system severely delayed or abolished the estrus cycle (Todd et al., 2007). GnRH neurons display IGF-1 receptors (Daftary and Gore, 2004). It is noteworthy that GnRH neurons also synthesize IGF-1 (Miller and Gore, 2001). The upregulation of IGF-1R in GnRH neurons in proestrous mice indicates that this growth factor signaling is operating in adult GnRH neurons of mice and that its effects upon GnRH neurons are gonadal cycle phase dependent. IGF-1 may derive from GnRH neurons and acting upon IGF-1R expressed by other GnRH cells; thus, it can contribute to synchronization of GnRH neurons prior to the GnRH surge. IGF-1 administration increased the frequency of mPSCs in GnRH neurons of metestrous mice, providing evidence for the responsiveness of the receptor to the ligand. Previous studies have shown the capability of IGF-1 to modulate Ca^2+^ channels in neuroblastoma cells (Kleppisch et al., 1992) and modify the electrophysiological properties of dorsal column nucleus (DCN) neuron in the brain stem (Nunez et al., 2003). Elucidation of the role of IGF-1 in the regulation of adult GnRH neurons requires further studies.

#### Growth Factor Receptor-Bound Protein 2 and 10

Both genes encoding these adaptor proteins were upregulated. GBR2 is known to bind to epidermal growth factor receptor (EGFR), whose coding gene was also affected by proestrus. The GRB 10 protein interacts with IGF-1 and IGF-2 receptors, as well with insulin receptor.

#### Nerve Growth Factor Receptor Associated Protein

In proestrus, Ngfrap1 gene was also upregulated, which codes for brain expressed X-linked protein (BEX3), whose role in the regulation of the GnRH system is obscure. It has been reported to regulate NGF-dependent survival and differentiation of neurons by enhancing trkA gene transcription (Calvo et al., 2015).

#### Epidermal Growth Factor Receptor

EGFR immunoreactivity is widely distributed in the hypothalamus including the OVLT region (Ma et al., 1994). It is expressed in both neurons and glial cells; however, GnRH neurons were found immunonegative for EGFR (Ma et al., 1994). In addition to EGF, the receptor binds transforming growth factor alpha. Our present finding shows that GnRH neurons of proestrous mice express an increased level of EGFR mRNA than those of metestrous mice.

#### Transforming Growth Factor Beta Receptor II

Immortalized GnRH neurons are regulated by transforming growth factor beta 2 (TGFB2) and contain TGFB receptor 2 mRNA (Messi et al., 1999). Exposure of the cell line to TGFB2 facilitated the release of GnRH and decreased the content of GnRH mRNA, indicating that this cytokine is a recognized regulator of GnRH cell functions.

In explants of the POA, about 40% of GnRH neurons were immunopositive for TGFBR2 (Bouret et al., 2004). Our present data strengthen the view about the regulatory role of TGFBR2 upon the GnRH system and provide evidence for the gonadal cycle-dependent expression of the receptor.

### Influence of Proestrus on the Expression Profile of Orphan G-Protein-Coupled Receptors

Proestrus differentially regulated nine orphan GPCRs in GnRH neurons. From this gene cluster, only GPR107 was upregulated; the rest underwent downregulation in proestrus. GPR107 is the putative receptor for neuronostatin, a hormone derived from pro-somatostatin (Yosten et al., 2012). Knockdown of GPR107 resulted in loss of responsiveness to neuronostatin. The receptor may also regulate the return of receptors to plasma membrane from endocytic compartments (Zhou et al., 2014). Neuronostatin increased the frequency of mPSCs in GnRH neurons in our study, which was blocked by G-protein inhibitor. In the hypothalamus, Samson et al. have reported (Samson et al., 2008) that neuronostatin depolarized paraventricular neurons in the presence of voltage-gated sodium channel blocker, TTX. GPR1 acts as a receptor for chemerin, which contributes to hypothalamic remodeling (Helfer et al., 2016). The significance of this signaling in case of GnRH neurons is unknown. GPR87 takes part in cell communication and is important in cancer pathology (Niss Arfelt et al., 2017). GPR18 is claimed to serve as receptor for endogenous lipid neurotransmitters, including the anandamide metabolite, *n*-arachidonyl glycine (Mchugh et al., 2010). GPR125 is an adhesion GPCR that is upregulated in traumatic brain injury (Pickering et al., 2008). GPR62 is expressed in the brain (Lee et al., 2001), but its function is still obscure. GPR183 was identified as an oxysterol receptor (Hannedouche et al., 2011). GPR4 is considered as a proton-sensing GPCR (Tomura et al., 2005). GPR88 is expressed in neurons. It regulates GABAergic medium spiny neurons in the striatum (Quintana et al., 2012).

GRK6 gene encodes GPCR kinase 6, which was markedly upregulated in proestrus. Its role is to disable the activated forms of GPCRs by phosphorylation. Among others, it takes part in the regulation of postsynaptic D1-like receptors (Gainetdinov et al., 2003).

### Other Signaling Mechanisms

#### Vomeronasal Receptors

All three vomeronasal receptor genes (Vmn1r172, Vmn2r-ps54, and Vmn1r148) showed downregulation in proestrus. The expression of vomeronasal receptors in GnRH neurons reflects the early stage of embryonic development in which GnRH neurons migrate from the vomeronasal organ toward the basal forebrain following the course of vomeronasal axons (Wray, 2010).

#### Platelet-Activating Factor Receptor

This GPCR binds the phospholipid platelet-activating factor (PAF). Proestrus evoked its downregulation. The signaling mechanism is associated with inflammatory processes. A recent report claims its role in the regulation of body weight and food intake (Li and Mcintyre, 2015). Its role in the central control of reproduction awaits clarification.

The regulation of the GnRH surge release is a highly complex mechanism. In rodents, the positive estradiol feedback has a marked effect on the excitability of GnRH neurons manifested in increased firing (Adams et al., 2018). It is also essential for tuning and synchronizing different neuronal inputs to GnRH neurons in proestrus including the potent kisspeptin input from the AVPV (Adams et al., 2018). Several classic neurotransmitter systems of the brain also alter their communication with preovulatory GnRH neurons as revealed by differential expression of their corresponding receptors (Vastagh et al., 2016). Furthermore, the changing hormonal milieu heavily influences the expression of major voltage-gated ion channel genes in GnRH neurons (Vastagh et al., 2019).

The explored neuropeptide/growth factor/orphan GPCRs are differentially regulated in GnRH neurons in late proestrus when the cells shift their operation mode to a higher level of activity. It is achieved by neuronal plasticity; and probably dozens of neurotransmitters, neuropeptides, and growth factors support simultaneously the achievement of this transient process. The clarification of the exact role of the identified novel modulatory systems requires further functional studies in the future.

## Data Availability Statement

The datasets presented in this study can be found in online repositories. The names of the repository/repositories and accession number(s) can be found at: https://www.ncbi.nlm.nih.gov/geo/, GSE66806.

## Ethics Statement

The animal study was reviewed and approved by Animal Welfare Committee, Institute of Experimental Medicine (Permission Number: A5769-01).

## Author Contributions

CV designed and performed the experiments and analyzed the data. VC and IF implemented the slice electrophysiological experiments. NS carried out the bioinformatics analysis of the microarray data. ZL designed and supervised the project and wrote the manuscript. All authors contributed to the article and approved the submitted version.

## Conflict of Interest

The authors declare that the research was conducted in the absence of any commercial or financial relationships that could be construed as a potential conflict of interest.

## Abbreviations

Acvr2b: activin receptor IIB
Adipor1: adiponectin receptor 1
Amhr2: anti-Mullerian hormone type 2 receptor
Cckbr: cholecystokinin B receptor
Ednrb: endothelin receptor type B
Egfr: epidermal growth factor receptor
Fgfr1: fibroblast growth factor receptor 1
Gpr1: G-protein-coupled receptor 1
Gpr107: G-protein-coupled receptor 107
Gpr125: G-protein-coupled receptor 125
Gpr18: G-protein-coupled receptor 18
Gpr183: G-protein-coupled receptor 183
Gpr4: G-protein-coupled receptor 4
Gpr62: G-protein-coupled receptor 62
Gpr87: G-protein-coupled receptor 87
Gpr88: G-protein-coupled receptor 88
Grb10: growth factor receptor-bound protein 10
Grb2: growth factor receptor-bound protein 2
Grk6: G-protein-coupled receptor kinase 6
Igf1r: insulin-like growth factor I receptor
Mc3r: melanocortin 3 receptor
Ngfrap1: nerve growth factor receptor (TNFRSF16) associated protein 1
Nmbr: neuromedin B receptor
Nmur1: neuromedin U receptor 1
Npr3: natriuretic peptide receptor 3
Prokr1: prokineticin receptor 1
Ptafr: platelet-activating factor receptor
Rtn4r: reticulon 4 receptor
Sctr: secretin receptor
Tgfbr2: transforming growth factor, beta receptor II
Vmn1r148: vomeronasal 1 receptor 148
Vmn1r172: vomeronasal 1 receptor 172
Vmn2r-ps54: vomeronasal 2, receptor, pseudogene 54.
